# A critical analysis of gender-based violence reporting and evidence building applications (GBVxTech) for capturing memory reports

**DOI:** 10.3389/fpsyg.2023.1289817

**Published:** 2024-01-18

**Authors:** Laura M. Stevens, Tia C. Bennett, Jessica Cotton, Sarah Rockowitz, Heather D. Flowe

**Affiliations:** ^1^School of Psychology, University of Birmingham, Birmingham, United Kingdom; ^2^School of Psychology, Cardiff University, Cardiff, United Kingdom

**Keywords:** gender-based violence, #MeToo, mobile applications, police interviews, rape, sexual violence

## Abstract

**Introduction:**

Gender-based violence (GBV) is under-reported to the authorities owing to the stigma, shame, and fear of reprisal that surrounds these crimes. To address this, there has been an influx of technologies, including mobile phone and online applications that allow victim-survivors (hereafter, victims) to document and report GBV (hereafter referred to as GBVxTech). We critically analysed the extent to which GBVxTech applications align with the scientific knowledge base on gathering accounts of crimes from victims and witnesses.

**Methods:**

We identified 41 reporting and evidence building applications from around the world but found many (*n* = 19) were no longer accessible. A total of 13 applications met the study criteria and were available for download. We evaluated each application on how well its design and features align with established minimum best practice standards for gathering complete and accurate accounts from witnesses and victims, such as the pre-interview instructions (e.g., setting ground rules), questioning approach (e.g., using open-ended questions), and the adequacy of security features (e.g., password protection).

**Results and Discussion:**

We found most applications employ open questions, encourage victims to report information in an independent voice, and seek to elicit information pertinent to a criminal investigation. None of the applications use leading questions. However, most applications do not establish ground rules, and many use forced-choice questions, do not time stamp the information gathered, or document when users change their answers. Many applications have limited security features, potentially compromising users’ safety. Further, some applications do not provide information about how to use the app, an informed consent procedure, or data usage information. We discuss the findings and offer recommendations for future GBVxTech development.

## Introduction

Gender-based violence (GBV) is any harmful act perpetrated against an individual or group due to their gender, and encompasses domestic, sexual, psychological, financial, and digital violations, as well as female genital mutilation, human trafficking, and child marriage (United Nations, n.d.). GBV affects men, women, and children worldwide, with global estimates finding that one in three women over the age of 15 have experienced intimate partner violence or non-partner sexual violence at least once during their lifetime (World Health Organization, 2021), and one in three men in the United States having experienced some form of sexual violence, intimate partner violence, or stalking within their lifetime (Smith et al., 2018). Furthermore, the actual prevalence of GBV may be higher considering that many people do not disclose their victimisation (World Health Organization, 2003) due to stigma, shame, and fear (Shaibakova, 2020). Moreover, of all people who experience GBV, fewer than 40% seek any form of legal or medical assistance (United Nations, 2015), and even fewer will have their case prosecuted (Home Office, 2021).

Given global underreporting of GBV, the #MeToo movement was founded initially to support women and girls of colour who experienced sexual violence (Me Too Movement, 2023) and to highlight how gender, race, and other inequalities intersect “to produce unique experiences of violence” (Imkaan, 2019, pp. 3). The #MeToo movement later became a channel for victim-survivors (hereafter, victims) of sexual violence to disclose incidents on social media. These social media disclosures are revealing not only the widespread prevalence and nature of GBV but are also ushering in a burgeoning of mobile phone and online applications for gathering information about GBV incidents, hereafter referred to as *GBVxTech* (World Bank Group, 2019). Victims, irrespective of gender, can use these applications to get help with accessing vital support services (e.g., medical and legal facilities) and to document incidents anonymously or confidentially, either in real-time or retrospectively. These digital data could potentially be admitted as evidence at trial in the United States (Miller, 2022), the United Kingdom (Hollie Guard, 2021), and Australia (Paterson, 2018).

### Benefits of GBVxTech

According to research, sexual assault victims and their support providers want an alternative reporting system, such as mobile phone applications and online reporting platforms, in addition to formal criminal justice reporting methods (Heydon et al., 2023). There are many reasons for this. First, many applications and websites allow victims to report incidents in real-time and store their report (e.g., iWitnessed), which is crucial in situations where victims cannot report to the authorities. There may be no police station nearby for the victim to access support, such as in rural areas and in low- and middle-income countries (see Smith et al., 2019), or victims may not be able to escape their attacker and make a report, such as in domestic violence cases. Compared to requiring formal police interviews, giving victims the flexibility to report sexual assault through informal or anonymous channels can reduce barriers and increase overall reporting rates (Heydon et al., 2023). GBVxTech also stores reports for potential future use in criminal proceedings if victims choose to formally report later (Paterson, 2018).

Second, GBV victims frequently delay reporting incidents to law enforcement (Read and Connolly, 2007; Loney-Howes et al., 2022), which results in missed opportunities to promptly gather forensic evidence and victim accounts (i.e., memory evidence). Memory strength for the crime decreases with time (Ebbesen and Rienick, 1998; Gabbert et al., 2009, Stevens et al., 2022). With GBVxTech, victims can document a GBV incident soon after it occurs. Research has found that an early initial free recall attempt can maintain the accuracy and completeness of an individual’s account over time if victims and witnesses are interviewed following recommended practise (e.g., Penrod et al., 1982; Wixted and Ebbesen, 1997; Ebbesen and Rienick, 1998; Gabbert et al., 2009), including in sexual offence cases (Stevens et al., in prep.). Further still, immediate self-documentation can preserve memory accuracy over time (Gabbert et al., 2022; Stevens et al., 2022), and reduce susceptibility to misleading post-event information (Gabbert et al., 2012). This is particularly important in GBV cases because the victim’s account is often the primary, if not only, source of evidence (Kebbell et al., 2007). Actual or perceived gaps or inconsistencies in victim accounts can diminish prosecution odds, as officials may think gaps and inconsistencies signal that the victim lacks credibility (Freyd, 2004). Thus, timely documentation not only maintains accuracy (Ebbesen and Rienick, 1998; Gabbert et al., 2009) but also can serve to indirectly maintain the victim’s credibility (see Westera et al., 2011, 2013).

Third, some victims may prefer to use GBVxTech over contacting the police because the technology allows them to anonymously report the incidents to help law enforcement prevent future crimes. One example of an anonymous reporting app is SafeTTC (n.d.), where individuals can report incidents occurring in public spaces (e.g., public transport) to provide key information about local hotspots. JDoe (n.d.) and Callisto (n.d.) are also apps that allow anonymous reporting, and both use algorithms to monitor when multiple reports refer to the same perpetrator for purposes of identifying serial perpetrators.

Finally, GBVxTech may be used by those who wish to seek support services (Heydon et al., 2023) or who intend to seek legal redress through civil action rather than criminal prosecution. For example, some apps (e.g., JDoe and Callisto) provide victims with the opportunity to be contacted by a lawyer or legal advisor to discuss their options.

### Minimum best practice principles and GBVxTech

The completeness and accuracy of victim accounts are largely dependent on adherence to minimum best practice principles for face-to-face interviews (e.g., Read et al., 2009; Brubacher et al., 2014). These principles were recently extended to self-administered written interviews that use open questions and free recall formats (Gabbert et al., 2009, 2022). These principles set a minimum standard for interviews, and include establishing rapport and trust, providing narrative practise, setting ground rules, using open questions, and allowing for the victim’s account to be appropriately documented (i.e., in the victim’s own words, or via their ‘independent voice’; Powell et al., 2005). It is also crucial that interviews adhere to principles that protect the victim’s human rights (Murad Code, 2022). Since the emergence of GBVxTech is recent, there has been little consideration of how these technologies might adopt these evidence-based principles. Below, we discuss the core principles of interviewing, and how they might be applied to GBVxTech.

#### Rapport building and trust

Rapport building is the process of establishing a relationship with another individual, and its use during an interview allows people to feel more at ease (Vallano and Schreiber Compo, 2015). Establishing rapport can make victims feel more comfortable to disclose information (Fisher and Geiselman, 1992). When an interviewer establishes rapport with a victim, it promotes a feeling of comfort, whereby a victim feels safe and relaxed to discuss their experiences (Patterson, 2011). Interviewers can foster this environment by ensuring the victim feels believed (Patterson, 2011), displaying empathy (Greeson et al., 2014; Kim et al., 2020), and by being personable with the victim (e.g., sharing a personal detail/story separate to the event to be discussed within the interview; Greeson et al., 2014). Rapport building has been found to increase the accuracy of the victim’s account (Kieckhaefer et al., 2014; Vallano et al., 2015), and increase the probability that victims who wish to make a formal complaint will carry through and complete the reporting process (Brooks and Burman, 2017).

Rapport building is essential in dyadic person-to-person interviews for sexual assault (Westera et al., 2016). Rapport is established through dynamic individual exchanges (see Abbe and Brandon, 2013; Gabbert et al., 2021, for reviews), whereas apps feature structured questioning and inflexible interaction. Undeniably, it is harder to implement rapport building techniques using technology compared to face-to-face contexts (Meijer et al., 2021). Non-verbal communication (eye contact, open body language etc.) and verbal communication (affirmative responses, comforting the victim during disclosure) are methods used in rapport building (Abbe and Brandon, 2013), and these cannot be simulated in GBVxTech applications. Trust, however, is one component within the definition of rapport (Neequaye and Giolla, 2022) that could be achieved in a non-dyadic context (Meijer et al., 2021). Examples include ensuring the technology clearly conveys the purpose of data collection, reassuring users with respect to data security, and obtaining informed consent regarding data storage and usage (Liu, 2018; Obada-Obieh et al., 2020). By establishing trust, users may feel more comfortable sharing information (Abbe and Brandon, 2013; Gabbert et al., 2021), which is a fundamental reason for establishing rapport.

Another critical component of helping victims to feel more comfortable with the interview experience is narrative practice. This allows the interviewee to practise recalling a neutral or positive episodic event before they provide information about the crime (Roberts et al., 2004; Lyon et al., 2014; Yi and Lamb, 2018). This is beneficial because it gives victims the opportunity to familiarise themselves with the style of questioning that will be used during the interview (Roberts et al., 2011), which in turn increases the number and accuracy of details disclosed during subsequent recalls (Sternberg et al., 1997; Price et al., 2013). Beyond increasing memory recall accuracy and completeness, narrative practise has also been found to help victims feel more comfortable during the interview by improving their understanding of the interview process (Brubacher et al., 2020). Narrative practise could be implemented within GBVxTech by asking victims to freely recall a positive or neutral event before beginning their report.

#### Ground rules

Ground rules are clear, simple instructions given to the victim that establish what to expect during the interview (Powell et al., 2005). These instructions enhance memory reporting during the interview and help to manage interviewee expectations (Fessinger et al., 2021). Key ground rules include encouraging the interviewee to say ‘I do not know’ when they do not know an answer to a question, and correcting the interviewer if the interviewer does not accurately understand what the interviewee has said (Ridley et al., 2012). In addition to managing expectations, encouraging ‘do not know’ responses increases the accuracy of memory reports (Scoboria and Fisico, 2013). Although most of the research about ground rules has focused on child interviewees, it is also useful with adults during investigative interviews (Ali et al., 2020) and in lineup tests (Weber and Perfect, 2012; Wells et al., 2020). These research studies illustrate the importance of ground rules; but it is unclear to what extent these rules have been applied in the context of GBVxTech.

We were particularly interested in whether the applications we found would allow users to indicate when they do not know an answer to a question, or instead either require users to provide information or allow them to leave questions blank. Requiring users to provide information that they do not explicitly remember is problematic should the case progress to investigation. Requiring responses can introduce inaccuracy, inconsistency, or uncertainty in accounts. Allowing ‘do not know’ or ‘do not remember’ options protects against false information whilst signalling when users truly lack memory for certain details.

#### Independent voice

During an interview, it is essential that the victim’s voice is heard and not influenced by other information. This can occur if the victim overhears information provided by other witnesses (Gabbert et al., 2003) or via information shared by the interviewer (Loftus and Palmer, 1974). It is also particularly useful for interviewers to encourage a free recall account via open-ended prompts at the beginning of the interview before asking specific questions to ensure that the victim’s initial account is given without any influence from the interviewer (Fisher and Geiselman, 1992; Powell, 2002; Ministry of Justice, 2022). Additionally, interviewers should not interrupt the victim whilst they provide their account, because this can discourage the victim from taking an active role in the interview or may break the rhythm of their recall, which could result in their not reporting details that they otherwise would have remembered and reported and/or damage their independent voice (Fisher and Geiselman, 1992). Further, accounts should be recorded verbatim, without bias or opinion from the interviewer (Powell, 2002), in the victim’s native language to reduce cognitive load and maximise accuracy (Raver et al., 2023), and in a format that is simple and accessible for the victim (e.g., typed/voice recorded, Gabbert et al., 2009). Despite evidence that this principle is crucial for obtaining an accurate report from victims, the extent to which independent voice is maintained in the context of GBVxTech remains to be seen.

#### Open questions

Open questions are essential during a police interview. Open questions are used to elicit unrestricted answers and allow the interviewee to give a free narrative account of events (Ministry of Justice, 2022). Due to the nature of open questions, they have a broad focus and do not dictate what information the interviewee should be reporting (Powell and Snow, 2007), which enables interviewees to respond freely. Open questions are beneficial because they result in more detailed and accurate responses than yes/no and forced-choice questions (Oxburgh et al., 2010; Westera et al., 2011). Furthermore, open questions reduce the risk of the interviewer influencing the victim’s response by imposing expectations or bias (i.e., avoiding leading questions, Milne and Bull, 2006). Recommended practice entails asking open questions that have the least possible influence on memory reporting, thereby enabling victims to provide an independent account (Milne and Bull, 1999; Heydon and Powell, 2018). Given the importance of open questions in obtaining detailed and accurate statements, we were interested in the extent to which GBVxTech utilises them. Whilst open questions are considered best practice, most interviewers use specific questions (Powell et al., 2005). Specific questions, including who/what/when/where questions, yes/no questions, and forced choice questions (Benson and Powell, 2015) encourage the interviewee to answer with a single word or detail, and this tends to limit the amount of information elicited from the interviewee (Lyon, 2014). Therefore, we also examined the use of specific questions in reviewing the applications.

#### Human rights principles

Our analysis was also inspired by fundamental human rights standards and principles regarding respect for the life, dignity, and privacy of interviewees, as outlined in the Murad Code (2022) and emphasised by Juan Mendez, the UN Special Rapporteur on Torture (Mendez and Areh, 2021). The Murad Code, named after Nobel Peace Prize winner Nadia Murad, outlines ethical standards for interviewing sexual violence victims, including obtaining informed consent, protecting their privacy and safety, and avoiding re-traumatisation. These human rights principles also extend to digital evidence gathering through GBVxTech, which is key in humanitarian crises where users face heightened vulnerability (Hankins, 2019). However, the principles also apply more broadly given the sensitive nature of sexual violence evidence, regardless of context.

### Evaluation of GBVxTech

GBVxTech can be a vital tool for GBV protection, prevention, and response (Eisenhut et al., 2020). For example, a report from one mobile app, SafeTTC, aided in the arrest of an individual who was already wanted as a suspect for a separate assault (Spurr, 2017). Furthermore, globally, 83% of individuals own a smartphone (Statista, 2022), making smartphone-based technologies that record and report cases of GBV accessible and available to many members of the public, especially in the Global North. As such, GBVxTech is potentially a powerful tool to address the underreporting of GBV and strengthen the evidence base necessary for successful prosecutions. Whilst GBVxTech holds promise, its use requires diligence as improper use risks harm. Our study critically evaluates GBV reporting apps intended to aid victims in alerting authorities. Though well-intentioned, these tools require appraisal to realise potential benefits.

In the only research of its kind to date, Eisenhut et al. (2020) systematically reviewed mobile health intervention apps that address violence against women. The authors classified apps into five categories: emergency (e.g., send emergency alerts to selected contacts), avoidance (e.g., avoid potential incidents), education (e.g., increase knowledge), reporting and evidence building (e.g., report an incident during or after it occurs), and supporting (e.g., used to provide support for victims). The authors found that just under half of the apps (47%) had the primary function of offering immediate help in emergencies, such as alerting emergency contacts or the nearest police station before, during, or soon after an incident. Furthermore, only 14% of apps were categorised as reporting and evidence building apps, although the prevalence of these apps was found to be increasing over time. The review also revealed that there is a growing number of education and supporting apps, which suggests that there may be a shift towards building apps that support individuals *after* the incident rather than during an emergency.

We extend the research conducted by Eisenhut et al. (2020) by analysing the extent to which applications in the ‘reporting and evidence building’ category both employ best practice principles for eliciting accurate and reliable information from victims and take steps to protect the victims who use them from further harm. We focus on this category because this type of GBVxTech was developed for the express purpose of recording information about the crime to share with law enforcement should the victim decide to report the crime(s) to the authorities. Altogether, given the ethical, legal, and social ramifications of GBVxTech, there is a need to better understand (1) how these applications elicit GBV accounts from users, and (2) the steps being taken by GBVxTech developers to protect users and their data.

### The current study

The core best practice principles discussed above are vital in gathering accurate and detailed accounts in face-to-face and written interviews. However, GBVxTech only recently emerged and therefore little is known about whether best practice principles are being adapted and implemented in this technology. We address this gap by critically analysing GBVxTech reporting apps that are currently in use and offering recommendations for how face-to-face interview principles may be adapted within a virtual context.

Our research has two objectives. Firstly, to evaluate the extent to which the best practice principles have been implemented in GBVxTech, and secondly, to draw on our findings to stimulate research and policy development so that the potential benefits of GBVxTech can be better realised.

## Methods

### GBVxTech identification

#### Identification plan

**Figure 1:**
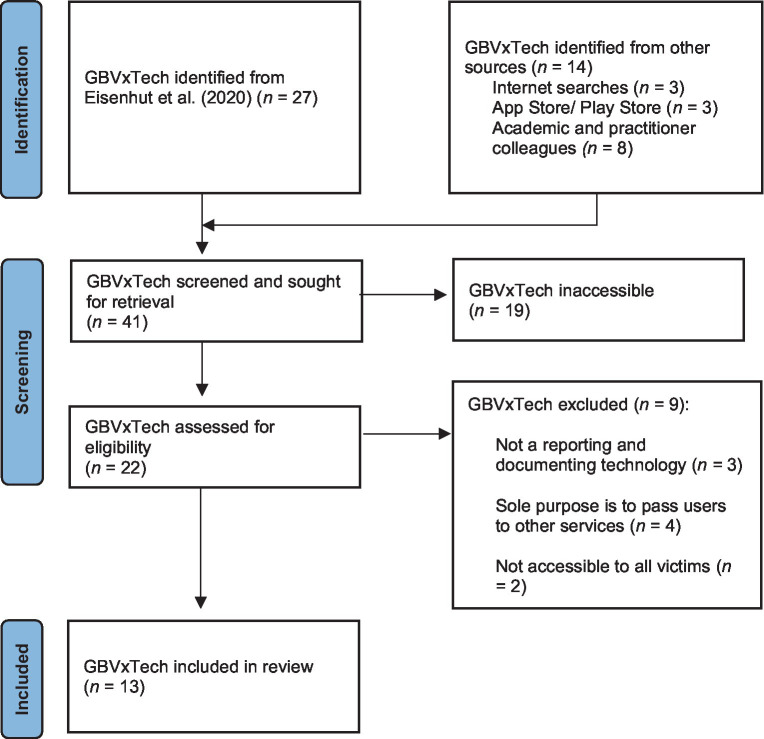
GBVxTech identification PRISMA diagram.

This review followed the PRISMA framework (Page et al., 2021) when searching for GBVxTech and selecting our final sample. To identify all relevant GBVxTech platforms, keyword searches were conducted on Google Scholar, Google, Twitter, Play Store (Android), and App Store (iOS) for the following search strings: ‘gender-based violence + reporting and evidence-based app*’ (to capture all denominations of ‘app’, ‘apps’, ‘application’, and ‘applications’); ‘gender-based violence + reporting app*’ and ‘gender-based violence + reporting tech*’ (to capture all denominations of ‘technology’). Additionally, ‘gender-based violence’ was replaced with ‘sexual violence’, ‘sexual assault’, and ‘domestic violence’. To ensure we located all possible GBVxTech platforms for inclusion within our review, we also completed a reference and citation search on the paper of Eisenhut et al. (2020) and held discussions with academic and practitioner colleagues (see Figure 1).

#### Selection criteria

The GBVxTech platforms included in this study were those that document reports of GBV specifically. The GBVxTech accessed were filtered based on our exclusion criteria, with any GBVxTech that met one or more of our exclusion criteria being removed from the final review. Our exclusion criteria included: (i) if the GBVxTech only signposted victims to support services, crowdsourced crime information, or provided educational content but did not allow them to document a specific incident as this would not preserve their memory evidence for use in criminal justice proceedings; (ii) if researchers were unable to access the GBVxTech using a United Kingdom smartphone or laptop, a VPN, or via discussions with the creators, as we were unable to review the GBVxTech in its entirety; and (iii) if GBVxTech was not accessible to all service users (e.g., only accessible to young people aged 10–24).

### Data extraction

The evaluation checklist for face-to-face interviews used by Benson and Powell (2015) was adapted to make it relevant to GBVxTech. Alongside the original criteria, another evaluation criterion exclusively related to technology (‘GBVxTech characteristics’) was added based on recommendations from Martínez-Pérez et al. (2015). The adaptations were discussed and finalised by the research team to produce the modified checklist that was used for data extraction (see Table 1 for the modified evaluation criteria checklist). Data extraction was completed by conducting a mock report within each application.

**Table 1:** Evaluation criteria checklist.

Best practise principles criteria		Definitions and examples
Questioning strategies	Open questions	Questions that allow a detailed responsee.g., “*Tell me what happened?*”
	Specific questions	Questions that request a particular detail through forced-choice, yes/no prompts or who/what/where/when/why/how questions e.g., “*what did the perpetrator look like?*”.
		Leading questions	Questions that suggest a particular answer and may introduce information that the victim never mentioned e.g., “*what did the male look like?*” (*When the victim never mentioned the gender of the perpetrator*).
	Responding methods		Different ways to answer a question e.g., *free recall textbox, drop-down menu* etc.
		Adherence to the interview protocol	Trust	Building a relationship with the victim to make them feel more comfortable during the interview. Examples of trust building methods within GBVxTech include: *Asking for consent* (e.g., *via a pop-up*) *Giving an introduction to the app/website* *Explaining the purpose of the GBVxTech*
	Narrative practice		Allowing the victim the opportunity to practise recalling a neutral or positive episodic event before they provide information about the crime *Narrative practice* (e.g., *practise questioning phase*).
	Ground rules		Ground rules are instructions given to the victim about what to expect during the interview. Examples of ground rules within GBVxTech include: *Reporting ground rules* *Ability to view completed report to clarify misunderstandings*
Independent voice	Allowing the victim to respond using their own words and experiences. Examples of this within GBVxTech include: *Access to more than one language* *Can upload voice notes or videos* *No interruptions during report* *Use of open questions* *Free recall methods of responding* (e.g.*, text boxes*) *Can victims view other victim’s reports?*	
Debrief	Giving support to the victim after they have completed the interviewe.g., *helplines, website links* etc.	
	Investigative questions		Investigation relevant details. *The identity of the offender* *The approximate time of the offence* *The location* *The offence type* *Possible witnesses* *Possible physical evidence*
GBVxTech characteristics	Data usage clarity	Did the GBVxTech explain what would happen with the victim’s data after providing a report? e.g., *data storage and usage statement.*
		Security options	Did the GBVxTech have security provisions? e.g., *password/pin protection.*

### Measures

The modified GBVxTech evaluation checklist adapted from Benson and Powell (2015) consisted of four themes: questioning strategies, adherence to the interview protocol, investigative questions, and GBVxTech characteristics (Martínez-Pérez et al., 2015). Each of the criteria on the evaluation checklist was either coded as ‘present’ (if there was at least one instance of the criterion) or ‘absent’ (if no element of the criterion was seen) within each evaluated GBVxTech platform.

### Reliability

Two researchers independently assessed all potential GBVxTech search results against the inclusion and exclusion criteria for final selection within the review. Any discrepancies were discussed with the senior author for final selection.

The same two researchers extracted information from the GBVxTech using the above evaluation criteria. Additionally, two blind coders evaluated around 60% of the GBVxTech for purposes of inter-rater reliability (*n* = 8). Overall, there were moderate to perfect levels of agreement (< 60% Kappa) on all evaluation criteria and discrepancies were discussed to yield the results presented below.

## Results

Table 2 provides a descriptive overview of each of the 13 GBVxTech platforms reviewed.

**Table 2:** GBVxTech description.

GBVxTech	Country	Affiliation	Purpose	Availability
SafeTTC	Canada	Elerts	To report harassment or safety concerns on transport	iOS & Play Store
iWitnessed	Australia	University of Sydney & UNSW	To collect memory evidence for violent incidents (e.g., domestic violence)	iOS & Play Store
Kharita: Harass Map	Egypt	Harass Map	To report sexual harassment and intervention, and to show the scope of the problem in Egypt on a map	Play Store and Online Website
Jdoe	United States	Ryan Soscia	To provide an anonymous platform to report sexual misconduct and to connect victims with legal professionals	iOS & Play Store
SV_Case Study	Kenya	Wangu Kanja Foundation	To allow victims to document their experiences and to monitor case progression along the criminal justice pathway	iOS & Play Store
Stop Sexual Harassment Video Recorder	United States	Safe Workplace LLC	To discretely collect audio and visual data on sexual harassment	iOS & Play Store
Bright Sky	United Kingdom	Hestia	To signpost support services and to document victim’s experiences	iOS & Play Store
Spot	United States	All Turtles	To report misconduct at work	AI Chatbot through Workplace
eyeWitness to Atrocities	United Kingdom	International Bar Association	To discretely capture photos and videos of atrocities.	Play Store
Hollie Guard	United Kingdom	Hollie Gazzard Trust	To gather real-time evidence of incidents, as well as provide alerts and journey tracking capabilities	iOS & Play Store
Callisto	United States	Callisto	To document assault and to match reports of individuals harmed by the same perpetrator	Website (need United States campus email)
DocuSAFE	United States	National Network to End Domestic Violence and Office on Victims of Crime	To document incidents of abuse and share with legal professionals	iOS & Play Store
Report & Support	UK	Culture Shift	To document bullying or harassment for students, staff, and visitors of UK Universities	Website

Table 3 illustrates how the 13 GBVxTech platforms performed on each of the criteria within the modified evaluation checklist. The results are discussed below, and implications for these results are explored in the discussion.

**Table 3:** GBVxTech evaluation results.

Questioning strategies												
	Safe TTC	iWitnessed	Kharita: Harass Map	Jdoe	MobApp	Stop Sexual Harassment Video Recorder	Bright Sky	Spot	eyeWitness to Atrocities	Hollie Guard	Callisto	DocuSAFE	Report & Support
Open question usage	x	x	x	x		x	x	x	x	x	x	x	x
Specific question usage	x	x	x	x	x			x			x	x	x
Leading question usage													
Open responding methods	x	x	x	x		x	x	x	x	x	x	x	x
Closed responding methods	x		x	x	x			x			x	x	x
Adherence to interview protocol												
	Safe TTC	iWitnessed	Kharita: Harass Map	Jdoe	MobApp	Stop Sexual Harassment Video Recorder	Bright Sky	Spot	eyeWitness to Atrocities	Hollie Guard	Callisto	DocuSAFE	Report & Support
Trust	x	x	x	x			x	x	x	x	x	x	x
Narrative practice													
Ground rules	x	x	x	x		x	x	x	x	x	x	x	x
Independent voice	x	x	x	x	x	x	x	x	x	x	x	x	x
Debrief		x	x				x				x	x	x
Investigative questions												
	Safe TTC	iWitnessed	Kharita: Harass Map	Jdoe	MobApp	Stop Sexual Harassment Video Recorder	Bright Sky	Spot	eyeWitness to Atrocities	Hollie Guard	Callisto	DocuSAFE	Report & Support
Identity of offender		x		x	x			x			x		
Time		x	x	x	x	x		x	x		x	x	x
Location	x	x	x	x	x			x	x		x	x	x
Offence type	x	x	x	x	x			x		x		x	x
Possible witnesses		x						x			x		
Physical evidence	x	x	x		x	x	x	x	x	x	x	x	
GBVxTech characteristics												
	Safe TTC	iWitnessed	Kharita: Harass Map	Jdoe	MobApp	Stop Sexual Harassment Video Recorder	Bright Sky	Spot	eyeWitness to Atrocities	Hollie Guard	Callisto	DocuSAFE	Report & Support
Data usage clarity	x	x	x	x	x	x	x		x		x	x	
Security options		x				x	x		x	x	x	x	

An x indicates that the specified GBVxTech platform included at least one feature of the relevant criterion.

### Questioning strategies

Regarding question types, 92% of the GBVxTech platforms reviewed used open questions at least once, 69% used specific questions at least once, and 0% asked leading questions. Furthermore, specific questions were the most used question type, and only 23% of technologies opened with an open-ended invitation such as, ‘[Tell me] What happened?’ instead of a specific question. We also found that whilst most of the GBVxTech used open questions at least once, only 38% of GBVxTech used solely free recall responding methods, e.g., using a textbox to enter the crime report. Additionally 62% of all GBVxTech incorporated closed responding methods, such as drop-down menus and multiple-choice questions.

### Adherence to interview protocol

Regarding trust, only 23% of GBVxTech asked users for informed consent, 52% provided an introduction to the app/website, 85% discussed the purpose and aims of the technology, and 0% provided an option for narrative practice. Additionally, whilst only 15% of the GBVxTech reported specific ground rules instructions (e.g., to state ‘I do not know’), 92% allowed victims to view the final report before submitting to clarify any misunderstandings within the report.

We also investigated whether the GBVxTech encouraged an independent voice and found that only 38% offered more than one language option, and that 62% allowed users to report using a voice note or video/photo feature. Furthermore, only one of the applications (Kharita: Harass Map) allowed victims to view other victims’ reports. We also found that only 46% of GBVxTech offered a debrief (e.g., signposting to psychological support or advice).

### Investigative questions

Since GBVxTech data could be used in legal proceedings, we assessed which details were collected by the platforms. We found that 38% asked for the identity of the offender, 77% asked for the approximate time of the offence and the location of the incident (either pinpoint on a map or write the location), and 69% asked for the specific offence type such as sexual assault or rape. Additionally, 23% of GBVxTech platforms asked about the presence of possible witnesses, and 85% enquired about potential physical evidence (e.g., forensic evidence, weapons, photos/documents/videos of injuries) to provide corroborating evidence.

### GBVxTech characteristics

All GBVxTech briefly discussed how the data will be used within their terms and conditions, but only 77% clearly explained where data will be stored and for what purposes it can be accessed (e.g., whether the data can be forwarded to police, whether data are solely saved on the GBVxTech app or institutional server, etc.).

We also wanted to investigate the security options available within the apps and websites and found that 54% of our reviewed GBVxTech utilised a security feature, such as a password, a quick escape button from the application if someone approaches, or a disguise function that makes the app appear to be another type of app (e.g., a weather app).

## Discussion

In this paper, we investigated the extent to which GBVxTech apps and websites adhere to best practice interviewing principles. Applying these principles can improve the accuracy of victim reports and ensure the methods and evidence are legally reliable should the victim decide to involve authorities. Further, we wanted to better understand how GBVxTech ensures the protection of users and their data.

Our review found that GBV apps *partially* follow best practices; some features align with key principles, but there is room for improvement. We will highlight well-implemented features, identify lacking areas, and recommend enhancements.

### Adherence to best practice

The GBVxTech included in our review adhered to several minimum standards of best practice for interviewing victims about GBV incidents. For example, most of the applications used open questions, which helps to ensure that the victim’s account is captured in their own words and not influenced by suggestive interview questions (Elmir et al., 2011). Furthermore, none of the applications included in our review used leading questions. Leading questions can decrease a victim’s credibility as they can elicit self-contradictions within the victim’s testimony (Andrews and Lamb, 2016). By avoiding leading questions, GBVxTech can gather accounts that make for stronger legal evidence.

We also found that 12 out of 13 of the applications allowed victims to view their final report before submitting. On one hand, this could be viewed as a positive aspect of GBVxTech, since it allows victims to amend any mistakes or misinterpretations before submitting. On the other hand, by editing the final report before submitting, it may be argued that the evidence has been contaminated since the report is technically no longer a first account, and this could have repercussions in later legal proceedings. Only one application (Kharita: Harass Map), allowed victims to view other victims’ reports. Whilst no GBVxTech should have this feature, it is reassuring that so few applications enable reading others’ reports. Allowing victims access to others’ accounts is unethical, poses psychological and safety risks, and can potentially compromise the quality and utility of the victim’s own report. Seeing other accounts could invalidate the victim’s own experiences and dissuade them from reporting if they believe other incidents seem ‘worse’ than theirs. Victims’ memories also risk contamination if they incorporate details from others’ reports into their own (see Gabbert et al., 2003). Moreover, making reports public, even anonymised, endangers victims by enabling identification through case details. The lack of this feature across the applications we reviewed is positive, but technology creators must remain vigilant against its inclusion given the potential harms.

To address language and writing proficiency differences across users, we hoped that GBVxTech would incorporate a voice note or video feature to allow victims to report in their own words. We found that around 60% included this feature. This is crucial as it allows victims to disclose using their preferred method (text, voice note, video). In addition to addressing language barriers, voice note and recording facilities may increase user satisfaction and thereby people’s willingness to engage with the application and disclose information. User feedback on the Self-Administered Interview indicated many people prefer typing or recording answers over using paper and pen, because it is easier, more practical, and offers users greater flexibility (Gabbert et al., 2009).

The GBVxTech platforms we reviewed also effectively applied the principle of investigative questioning. Most of the applications asked investigative questions such as the identity of the offender, the time/location of the offence, offence type, potential physical evidence, etc. Since reports collected via GBVxTech may be used in later legal proceedings, it is crucial that these applications do not miss opportunities to collect investigation-relevant information. Whilst the inclusion of investigative questions in GBVxTech is positive, it is important to note that most applications used forced-choice closed response formats for these questions. Closed-ended questions can limit the level of detail obtained (Oxburgh et al., 2010) and constrain the user’s independent voice. Employing more open-ended response formats could elicit richer details from users in their own words.

All the GBVxTech platforms we reviewed explain in their terms how user data will be used. Furthermore, 77% clearly explain where user data are stored, such as whether the data are stored locally on the application. Ideally, all GBVxTech should explicitly detail data storage and access. This enables informed consent, as victims are able to actively understand how their data are being used (Heydon et al., 2023) regardless of the data protection rules in a given jurisdiction.

### Limitations of current GBVxTech

Whilst not all minimum standards of best practice in face-to-face interviewing are feasible within a digital format (e.g., verbal communication tactics to build rapport), some principles that would be simple to adapt are often missing (e.g., open questions, narrative practice, and ground rules).

Firstly, we found that only 23% of GBVxTech prompted the user for information starting with an open-ended invitation such as, ‘[Tell me] What happened?’ This low figure is concerning because according to face-to-face interview guidelines, a free recall account should always be obtained before asking specific questions (e.g., Achieving Best Evidence guidelines in the United Kingdom, Ministry of Justice, 2022). This is encouraged because it allows victims to give a complete, uninterrupted account in their own words (Powell, 2002; Brewer and Williams, 2017). Furthermore, more than half of all GBVxTech used closed responding methods, such as drop-down menus and multiple-choice questions. This response style raises numerous issues; firstly, these forced-choice methods limit the response options available, and therefore may not list the victim’s desired response option. As a result, victims may be forced to select an option that does not accurately capture their independent voice and experiences, or they may choose not to continue reporting if they feel their experience ‘does not count’ within the options provided. Secondly, since the victim’s account is limited to preselected response options, they cannot describe their experience in their own words. Forced-choice formats decrease the level of detail in reports, potentially reducing evidence quality for investigators. An open response format would also let victims rehearse details about the incident in their own words, strengthening their memory and any subsequent accounts they provide to legal officials.

We found that none of the GBVxTech employed narrative practice. Sternberg et al. (1997) found that children who practised providing a detailed account of a positive or neutral non-abusive episodic memory in the introductory phase of the interview gave more detailed narratives in the later recall phase, demonstrating the importance of practising a separate episodic recall prior to being interviewed about the event. Thus, GBVxTech can increase victim comfort and the level of detail provided by employing narrative practice. Our results also showed that most of the GBVxTech did not include ground rules instructions. This is problematic because, as mentioned previously, ground rules are useful for both adults and children during investigative interviewing to obtain both detailed and accurate accounts (Lyon, 2014; Ali et al., 2020). Future GBVxTech should consider prioritising the introductory phase of the application before a victim begins their report. During the pre-interview phase, trust in the application could be built and expectations set through ground rules and narrative practice, which will promote more detailed and accurate responses.

Another issue we found is that most applications allow users to report in only one language. The limited language options could be damaging to the victim’s independent voice. They should be able to report in their first language to capture the most accurate account (Raver et al., 2023). Moreover, if a victim is forced to report using a language in which they are not proficient, it could impact the accuracy of the report via errors in translation (Evans et al., 2019) or it could deter a victim from using the application.

Our analysis found gaps in adherence to human rights principles amongst the reviewed GBV technologies. Less than 25% requested informed consent from users, contrary to ethical investigative standards (Murad Code, 2022). Additionally, only 46% offered post-reporting support, advice, or referral to services. This lack of victim debriefing conflicts with knowledge that GBV elevates risks for mental health consequences like PTSD, depression (Tjaden and Thoennes, 2006; Campbell et al., 2009) and substance abuse (Kilpatrick et al., 1997). Best practices dictate that GBV reporting platforms should connect users with information and access to support given the empirical links between victimisation and psychological distress.

Finally, only around half of the GBVxTech we reviewed incorporated at least one security feature. This raises major ethical and safety concerns, particularly in cases where victims are reporting intimate partner violence when a partner could easily access their mobile phone or computer and retaliate (Freed et al., 2018). Therefore, security options (e.g., passwords or ‘quick escapes’) should be implemented in every app or website created for the purpose of documenting GBV incidents.

### Recommendations

Whilst the GBVxTech we reviewed utilise *some* of the best practice interview principles which have been scientifically developed and tested for interviews in criminal justice contexts, we found that many of the technologies diverge from recommended minimum best practise standards for obtaining accurate and complete accounts. Digital evidence, like text messages and video recordings, have been permitted in GBV prosecutions, establishing precedent for admitting documentation from technology platforms (Hlavka and Mulla, 2018; Glasbeek, 2021). However, our findings reveal gaps in incorporating research-based memory retrieval strategies and ethical issues. Given the investigative and legal potential of these tools, further research should examine how to optimise memory recollection, ensure data protection, prioritise user wellbeing, and better align the applications to human rights-based principles. With careful design, GBVxTech could play a significant role in empowering victims whilst advancing just legal outcomes. However, additional interdisciplinary work is needed to actualise that potential responsibly and ethically.

GBVxTech should obtain informed consent, notifying users how data will be utilised, whether and how data can be withdrawn, and outlining plans if the application is discontinued (Martínez-Pérez et al., 2015; Heydon et al., 2023). Our analysis found that around half of the applications we identified were no longer accessible, raising questions about what happened to users’ data following discontinuation. Data loss risks accountability and can compromise the investigation of incidents and prevention of future crimes. As application technology evolves rapidly, many apps become defunct due to lack of funding for updates. Developers should carefully consider long-term sustainability and have transparent data protocols in case of discontinuation. Releasing applications without data retention plans risks doing more harm than good if evidence is lost when platforms cease operation.

The potential discoverability of victim disclosures on GBVxTech raises important considerations for users and designers. First, users should be advised that the information they report could be utilised as evidence in legal proceedings. Providing users with this knowledge is an essential part of the informed consent process. Second, developers must recognise that design features, like forced-choice questions, can potentially introduce memory errors and inconsistencies, which may damage victim credibility. It is critical that applications gather accounts in victims’ own words through open questions and response formats. Forced-choice options risk introducing inconsistencies that could undermine victim credibility later if the victim has to clarify answers they gave to forced choice questions. Since GBV victim testimony already faces heavy scrutiny and credibility challenges (Kelly et al., 2005), preserving free narrative is imperative.

If accounts gathered via GBVxTech are entered into legal proceedings, data authenticity and chain of custody (i.e., documentation of the sequence of handling, transfer, and storage digital evidence) will become important issues. Criminal proceedings may involve examining application metadata or obtaining sworn statements on application use. If applications allow revising responses, originals should not be overwritten, but rather preserved alongside the revised information with timestamps. Digital evidence risks manipulation, and therefore steps must be taken to better ensure data authenticity. Asking the victim the reason why they made revisions may be beneficial, as changes may otherwise imply memory unreliability at trial (Liu, 2018).

Finally, GBVxTech has immense potential to serve victims globally, but only if it is purposefully designed for accessibility across needs, resources, and contexts. The applications we reviewed would benefit from incorporating accessibility features for users with visual or cognitive impairments, such as text-to-speech and interface customisation options. Apps should partner with disabled persons organisations to incorporate accessibility best practises both in design and safeguarding features. GBVxTech should also function online, across different data bandwidths (WiFi/5G/4G/3G), across operating systems (Android, iOS, etc.), as well as offline to allow for timely documentation. Enabling offline access allows all victims to record accounts as soon as possible, benefiting evidentiary quality. Further, accessible design and clear language are crucial for GBVxTech, which likely attracts diverse users. GBVxTech should avoid legal jargon and use plain language to ensure accessibility for users with varying knowledge of GBV, the law, and reporting procedures, all of which can vary depending on jurisdiction. Our suggestion to use plain language aligns with recommended best practices for face-to-face investigative interviews (Dando and Milne, 2009; Farrugia et al., 2019). Onboard dictionaries or definitions can further aid understanding, particularly for crime classification questions, as many individuals lack awareness of distinctions between crimes like sexual assault versus sexual harassment. However, only five of the applications we reviewed offered built-in definitions. Developing a more inclusive GBVxTech platform has immense potential to broaden access to justice globally, especially if designed intentionally for clarity and transparency through built-in support features and avoidance of context-specific terminology.

## Conclusion

The #MeToo movement that ushered in an era of online disclosure revealed the widespread prevalence of sexual violence as well as the many barriers to formal reporting. As we move forward, ethically designed GBVxTech platforms can provide a safe digital space for victims to document experiences if formal channels remain inaccessible or undesirable. However, to serve both victims and justice, evidence-based practices must be implemented to maintain the accuracy of the victim’s account for purposes of crime prevention and, should the victim elect to make a formal complaint, legal proceedings. By working across sectors with victims, law enforcement, service providers, and researchers, GBVxTech developers can fulfil the diverse needs illuminated by #MeToo, creating empowering technologies that ethically gather victimisation experiences whilst advancing systemic reforms.

## Data availability statement

The raw data supporting the conclusions of this article will be made available by the authors, without undue reservation.

## Author contributions

LS: Conceptualization, Data curation, Formal analysis, Investigation, Methodology, Supervision, Writing – original draft, Writing – review & editing. TB: Data curation, Formal analysis, Methodology, Project administration, Visualization, Writing – review & editing. JC: Conceptualization, Data curation, Formal analysis, Investigation, Methodology, Writing – original draft. SR: Conceptualization, Data curation, Formal analysis, Investigation, Methodology, Writing – original draft. HF: Conceptualization, Data curation, Formal analysis, Investigation, Methodology, Supervision, Writing – original draft, Writing – review & editing.
